# Management of acute diarrhea in adults in China: a cross-sectional survey

**DOI:** 10.1186/1471-2458-13-41

**Published:** 2013-01-16

**Authors:** Feng-Qin Hou, Yan Wang, Jun LI, Gui-Qiang Wang, Ying Liu

**Affiliations:** 1 Department of Infectious Diseases, Peking University First Hospital, No.8, XiShiKu Street, Xicheng, District Beijing, 100034, China; 2 Medial Department, Beaufour-Ipsen (Tianjin) Pharmaceutical Co., Ltd, Beijing, 100028, China

**Keywords:** Acute diarrhea, Adults, Cross-sectional survey, China

## Abstract

**Background:**

The aim of this study was to evaluate the management of acute adult diarrhea in China and assess adherence of clinical practice to national guidelines and 2012 World Gastroenterology Organization guidelines.

**Methods:**

A cross-sectional survey was carried out among physicians in 20 hospitals in two different areas of China (Beijing, 10; Shaanxi province, 10). Summary statistics were calculated for the overall study group and for each region. Between-region differences were assessed with χ^2^ or *t*-tests.

**Results:**

Data were collected for 800 patients (≥18 years; mean ± SD age 37.0 ± 16.3 years; 56.4% female). The mean ± SD time between diarrhea onset and visiting a diarrhea clinic was 2.4 ± 1.6 days; this interval was significantly shorter in Beijing than Shaanxi (2.0 ± 1.4 vs 2.8 ± 1.8 days, respectively; *p* < 0.001). Overall, 31.4% of patients self-medicated before visiting the clinic, most commonly with antibiotics. Routine stool examinations were ordered for 70.6% of patients, vibrio cholera stool culture for 57.5%, but non-vibrio bacteria stool culture for only 11.4%. Only 61.6% of patients received fluid and electrolyte therapy: 28.3% oral rehydration solution (ORS) and 33.4% intravenous fluids (even though only 13.8% needed). Antibiotics were the most common drugs (60.8%) and the most common antibiotics were fluoroquinolones, followed by aminoglycosides. Totally 51.3% of patients received irrational antibiotic treatment (unnecessary for 47.9%; indicated but not prescribed for 3.4%). After antibiotics, the most commonly prescribed drugs were dioctahedral smectite (59.3%); For Shaanxi compared with Beijing, less individuals received ORS (7.8% vs 48.5%,respectively; *p* < 0.001) and more received intravenous fluids (46.3% vs 20.5%, respectively; *p* < 0.001). Significantly more of the patients in Shaanxi province were administered antibiotics (64.5% vs 57%, respectively; p = 0.03), and more received intravenous antibiotics than Beijing (49.0% vs 27.0%, respectively; *p* < 0.001).

**Conclusions:**

Adherence to both national guidelines and 2012 World Gastroenterology Organization guidelines for the management of acute diarrhea in adult was limited among tertiary hospital physicians. The findings suggest nationwide education and effective health policies are needed to improve medical practice and reduce the unnecessary burden on the healthcare system.

## Background

Acute diarrhea in adults is one of the most common diagnoses in general practice, and it remains responsible for high morbidity rates around the world [1-4]. In south-east Asia specifically, diarrhea morbidity rates in adults are 0.3 episodes/ person-years and these rates have remained unchanged in the last 30 years [5]. More generally, in industrialized countries, the incidence of acute diarrhea in adults is estimated to be 0.5–2 episodes/person-years; the corresponding figure could be much higher in developing and underdeveloped countries [6]. In general, it is recognized that acute diarrhea is a major cause of childhood mortality in developing countries; however, adult mortality from diarrhea is also not uncommon, particularly among the elderly, in whom the case–fatality ratio is even higher than in children [7]. In the United States, children under 5 years, and adults aged 60 years or more, each comprised one-fourth of hospitalizations involving diarrhea, with the older group representing 85% of deaths [7].

Acute diarrhea is a distressing disease, even among adults. Although the episodes are usually brief and self-limiting, the incapacitating symptoms often drive patients to see physicians in search of relief. Regulatory medical authorities provide guidelines for the management of adult diarrhea, but how well physicians adhere to these guidelines has not been adequately assessed. To date, most studies of acute diarrhea have focused on children [8-12]; the few studies in adults have mainly addressed individual aspects of the management of acute diarrhea, such as antibiotic use [13,14]. The present cross-sectional study was conducted to evaluate comprehensively the management of acute diarrhea in adults and assess adherence to guidelines in China and 2012 World Gastroenterology Organization (WGO) guidelines.

## Methods

### Study population and setting

A cross-sectional survey was carried out in adult patients (defined as ≥14 years of age) with acute diarrhea in China between 16 May and 21 July 2011. In this survey, acute diarrhea refers to acute infectious diarrhea. Patients were considered to have acute diarrhea if they were passing at least three loose or watery stools in 24 h or passing at least one bloody stool (macroscopic observation revealed blood mixed up with feces or inseparable from the stool), and with an illness duration no longer than 14 days.

The survey was conducted in 20 hospitals (10 hospitals in Beijing and 10 hospitals in Shaanxi province). The location and size of the 20 hospitals were listed in Table 1. The Chinese capital of Beijing is a municipality under the direct control of the Central Government. It is located in the north of China, with an estimated population of more than 60 million permanent residents and migrant workers. Shaanxi province is an economically underdeveloped province in northwest China, with an estimated population of more than 40 million permanent residents and migrant workers. In most regions of China, patients with acute diarrhea are referred to specialist clinics, which are required by the Chinese government to monitor for cholera using dark field microscopy and stool culture. As primary care facilities are limited, these specialist clinics tend to be based in hospitals. In the present survey, participating hospitals were tertiary and public hospitals, most of which also serve as teaching and research centers for health professionals in addition to providing tertiary care. All hospitals had diarrhea clinics providing services only for adult patients; diarrhea clinics specifically for children were excluded from the survey. Each diarrhea clinic had between four and nine hospital physicians, all of whom were at least resident physicians.

**Table 1:** Features of participating hospitals by region

**Items**	**Beijing**	**Shaanxi**	**P**
Type of hospital*			
Teaching (Tier 3A)	9 (90%)	3 (30%)	0.0024
Teaching (Tier 3B)	1 (10%)	1 (10%)	NS
Non-teaching (Tier 3A)	0 (0%)	5 (50%)	0.039
Non-teaching (Tier 3B)	0 (0%)	1 (10%)	1.0
Location‡			
Capital of China or province	10 (100%)	7 (70%)	0.2105
Non-capital	0 (0%)	3 (30%)	
Physicians status			
Resident	12 (37.5)	20 (37.0)	0.966
Attending physician	15 (46.9)	15 (27.8)	0.072
Associated chief physician	3 (9.4)	10 (18.5)	0.253
Chief physician	2 (6.3)	9 (16.7)	0.162
Number of bed (means ±SD)	1151 ± 381	816 ± 451	0.0894
Total diarrhea patients/site (means ±SD)	2719 ± 1460	1241 ± 661	0.0124
Enroll Number/site	40	40	NS
Enroll rate § (%)(means ±SD)	2.2 ± 2.2	5.0 ± 4.3	0.0878
Response rate of questionnaire	100%	100%	NS

* All of hospitals are Tier 3 hospitals and no community hospital. ‡ All of 20 hospitals are located in cities and no rural. § Enroll rate = Enroll number/total diarrhea patients × 100%.

### Data collection

An anonymous, paper-based, descriptive questionnaire was used for collecting data. The questionnaire required physicians to report the characteristics of patients with acute diarrhea whom they had evaluated, as well as any tests and medications that they had ordered for that patient. All patients visiting diarrhea clinics with acute diarrhea during the study period and who agreed to participate in the survey were included. Patients were included only once in the study, irrespective of the number of repeat visits to the clinic. Each participating hospital was encouraged to complete 40 questionnaires.

The questionnaire comprised: demographic characteristics of patients (age, gender); onset and duration of diarrhea; frequency of stool passage per day; stool characteristics (watery, loose or bloody); presence of vomiting, fever and abdominal pain; self-medication; underlying diseases; physical examination of patients; presence and severity of dehydration (severity of dehydration according to 2012 WGO guidelines [6]); stool examination under light microscopy and microbiological investigations; pharmacological management of diarrhea (i.e. oral rehydration solution, ORS; antibiotics; probiotics; herbal medicine; and/or other therapeutic drugs).

### Ethics

The ethics committee in human research at Peking University First Hospital, Beijing, China, approved the study (EC approval letter number: 2011[322]). Informed consent was obtained from patients whose medical data were included in the study. Participation was voluntary and no financial incentives were given. Personal identifying information was not collected for practitioners or participating patients.

### Data analysis

There was no a priori sample size calculation for comparison between areas: however based on an a posteriori calculation assuming around 400 subjects in each region, such sample size gives the ability of detect standardized effect size of at least 0.2 for comparison based on quantitative data and assuming a power of 80% and a two sided alpha error of 5%. For binary outcome, it gives the ability to detect odds ratio higher or equal to 1.85 and rates ranging from 0.1 to 0.9 (again with power of 80% and alpha 2 sided 5%). Data were analyzed using SPSS software (SPSS/Pct, version 13.0; IBM Corp, Armonk, New York, NY, USA). Between-region differences were assessed for categorical variables with the χ^2^ test and for continuous variables with the *t*-test. Continuous variables are expressed as means ± SD with 95% confidence intervals (CIs); differences were considered statistically significant at *p*-values <0.05.

## Results

### Questionnaire completion and treating physicians

During the 9-week study, 800 questionnaires were completed (40 questionnaires/hospital) and 86 physicians participated in treating episodes of diarrhea. In the survey, 39598 patients presented to the participating hospitals with acute diarrhea, 800 of these patients were finally included by the physicians in the study. The enroll rate is 2.0%. Response rate to questionnaires is 100% in all of 20 hospitals. Most physicians were residents or attending physicians (72.1%), and there were no differences in the physician status between the two regions. The features of participating hospitals see Table 1.

### Clinical characteristics and self-medication

All of the 800 participating patients were Chinese, 56.4% were female and the mean age was 37.0 ± 16.3 years (median, 31 years; range, 18–87 years). The mean ± SD age of patients in Beijing was significantly lower than that in Shaanxi province (35.6 ± 14.8 vs 38.5 ± 17.5, respectively; *p* = 0.011).

**Figure 1:**
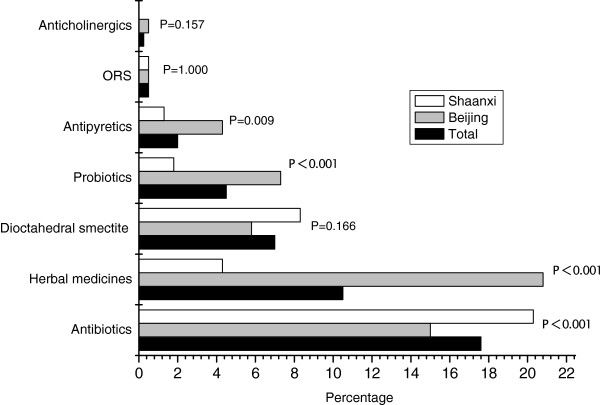
**Self-medication before visiting the diarrhea clinic. ***p*-values refer to comparisons between Beijing and Shaanxi province. ORS: oral rehydration solution.

Clinical characteristics and self-medication details are shown in Table 2 and Figure 1. The mean ± SD time between the onset of diarrhea and visiting a diarrhea clinic were 2.4 ± 1.6 days. In Beijing, the interval between onset of diarrhea and attendance at a clinic was significantly shorter than that in Shaanxi province.

**Table 2:** Clinical characteristics of diarrhea and laboratory tests ordered, overall and by region

	**Total (n = 800)**	**Beijing (n = 400)**	**Shaanxi province (n = 400)**	***p***
Complaints, n (%)				
Fever	261 (32.6)	162 (40.5)	99 (24.8)	<0.001
Abdominal pain	588 (73.5)	281 (70.3)	307 (76.8)	0.037
Vomiting	223 (27.9)	103 (25.8)	120 (30.0)	0.18
Stool characteristics, n (%)				
Watery	554 (69.3)	63 (65.8)	291 (72.8)	0.032
Loose	224 (28.0)	131 (32.8)	93 (23.3)	0.003
Blood	22 (2.8)	6 (1.5)	16 (4.0)	0.031
Frequency of stools/24 h, mean ± SD [95% CI]	6.2 ± 3.0	6.4 ± 3.2	6.0 ± 2.7	0.086
	[6.0; 6.4]	[6.1; 6.3]	[5.8; 6.3]	
Clinical dehydration, n (%)	136 (17.0)	72 (18.0)	64 (16.0)	0.451
Time from onset of diarrhea to visiting diarrhea clinic, n (%)				
<3 days	530 (66.3)	311 (77.8)	219 (54.8)	<0.001
3–7 days	252 (31.5)	86 (21.5)	166 (41.5)	<0.001
>7 days	18 (2.3)	3 (0.8)	15 (3.8)	0.004
Mean ± SD [95% CI], days	2.4 ± 1.6	2.0 ± 1.4	2.8 ± 1.8	<0.001
	[2.3; 2.6]	[1.9; 2.2]	[2.7; 3.0]	
Self-medication before visiting diarrhea clinic, n (%)	251 (31.4)	145 (36.3)	106 (26.5)	0.003
Laboratory tests ordered				
Routine stool examination	565 (70.6)	359 (89.8)	206 (51.5)	<0.001
Stool culture for vibrio cholera	460 (57.5)	400 (100)	60 (15.0)	<0.001
Stool culture for non-vibrio bacteria	91 (11.4)	34 (8.5)	57 (14.3)	0.01
Routine blood examination	445 (55.6)	205 (51.3)	240 (60.0)	0.013

Clinical dehydration: moderate-to-severe dehydration. *p*-values refer to comparisons between data for Beijing and Shaanxi province.

Overall, 251 (31.4%) patients had self-medicated before visiting a diarrhea clinic, with antibiotics being the most common drug (17.6% of patients). Significantly more individuals self-medicated in Beijing than in Shaanxi province. The most commonly used self-medications were herbal medicines in Beijing (20.8% of patients) and antibiotics in Shaanxi province (20.3%).

### Laboratory tests ordered by treating physicians

Routine stool and blood examinations were ordered for 70.6% and 55.6% of patients, respectively, in the overall study group. However, in Beijing, more patients had routine stool examination and fewer patients had routine blood examination than in Shaanxi province (Table 2). Overall, stool culture for vibrio cholera was ordered for 57.5% of patients, and stool culture for non-vibrio bacteria ordered for 11.4%. In Beijing, stool cultures for cholera were significantly more common and stool cultures for non-vibrio bacteria significantly less common than in Shaanxi province. No isolates were positive for vibrio cholera. A total of 79 of 91 stool cultures for non-vibrio bacteria had no outcome specified; of the 12 positive results, four were strains of *Shigella* spp, three were strains of *Escherichia coli*, one was a strain of *Salmonella* and four were strains of fungus.

### Interventions ordered by treating physicians

**Figure 2:**
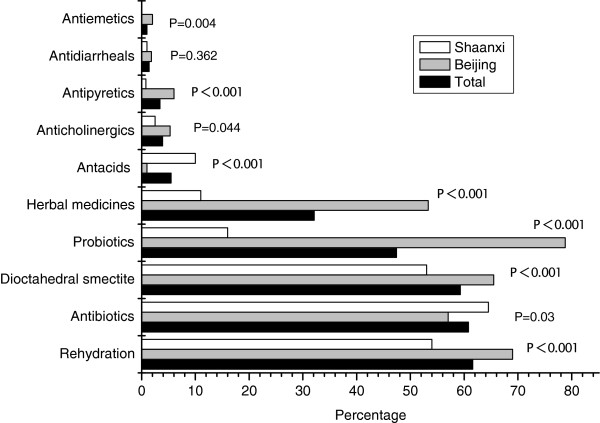
**Interventions ordered by treating physicians. ***p*-values refer to comparisons between Beijing and Shaanxi province.

The interventions ordered for the treatment of diarrhea are shown in Figure 2. The most common intervention was rehydration therapy (61.6% of patients). Antibiotics were the most common drugs (60.8%), followed by dioctahedral smectite (59.3%), probiotics (47.4%) and herbal medicine (32.1%). The order of drugs was similar in Shaanxi province, but, in Beijing, probiotics were the most common.

Details of the rehydration and antibiotic therapies ordered are shown in Table 3. ORS was ordered for 28.3% and intravenous fluid replacement for 33.4% (even though only 13.8% of patients presented with vomiting who unable to drink). In Beijing, significantly more patients received ORS and significantly fewer received intravenous rehydration than in Shaanxi province.

**Table 3:** Rehydration and antibiotic therapies ordered for diarrhea, overall and by region

	**Total (n = 800)**	**Beijing (n = 400)**	**Shaanxi province (n = 400)**	**P**
Fluid and electrolyte therapy	493 (61.6)	276 (69.0)	216 (54.0)	<0.001
Oral rehydration solution	226 (28.3)	194 (48.5)	31 (7.8)	<0.001
Intravenous fluid replacement	267 (33.4)	82 (20.5)	185 (46.3)	<0.001
Vomiting (unable to drink)	110 (13.8)	32 (8.0)	74 (18.5)	<0.001
Antibiotics	486 (60.8)	228 (57.0)	258 (64.5)	0.030
Two kinds of antibiotic combined	49 (6.1)	4 (1.0)	45 (11.3)	<0.001
Oral	181 (22.6)	120 (30.0)	61 (15.3)	<0.001
Intravenous	304 (38.0)	108 (27.0)	196 (49.0)	<0.001
Vomiting (unable to drink)	130 (16.3)	44 (11.0)	86 (21.5)	<0.001
Intramuscular	1 (0.1)	0	1 (0.3)	0.500

Data are number (%) unless stated otherwise. *p*-values refer to comparisons between data for Beijing and Shaanxi province.

**Figure 3:**
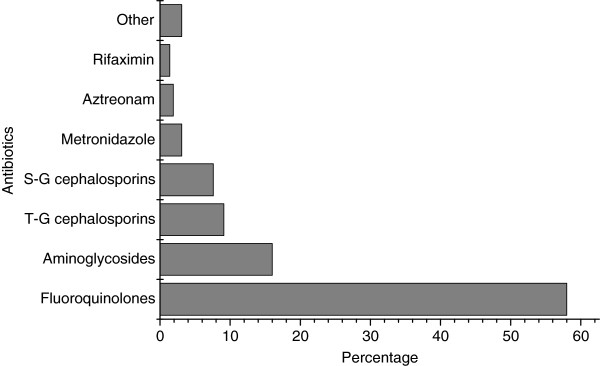
**Antibiotics prescribed by treating physicians.** T-G cephalosporins: third-generation cephalosporins, S-G cephalosporins: second-generation cephalosporins.

A total of 38.0% of patients received intravenous antibiotics even though only 16.3% presented with vomiting who unable to drink. Significantly more of the patients in Shaanxi province were administered antibiotics, including a combination of two antibiotics, and more received intravenous antibiotics than Beijing. A large number of different antibiotics were prescribed (Figure 3). Overall, the most common antibiotics were fluoroquinolones, followed by aminoglycosides. Of the fluoroquinolones, levofloxacin (70.2%) was the most common, followed by norfloxacin (18.1%). Among aminoglycosides, etimicin (98.7%) was used most commonly.

### Management of diarrhea cases with different diagnosis

The management for diagnosis of acute infectious diarrhea and acute bacterial dysentery were shown in Table 4. Of 727 cases with diagnosis of acute infectious diarrhea overall, 59.6% receive fluid and electrolyte replacement, the proportion was significantly lower in Shaanxi province than Beijing; 56.9% receive antibiotics therapy, the proportion was significantly higher in Shaanxi province than Beijing.

**Table 4:** Management by different diagnosis, overall and by region

**Diagnosis**	**Fluid and electrolyte replacement**	**Antibiotics**	**Dioctahedral smectite**	**Herbal medicine**	**Probiotics**
**Acute infectious diarrhea**					
Total (n = 727)	433 (59.6)	414 (56.9)	439 (60.4)	233 (32.0)	344 (47.3)
Beijing (n = 357)	242 (67.8)	185 (51.8)	242 (67.8)	191(53.5)	282 (79.0)
Shaanxi province (n = 370)	190 (51.4)	229 (61.9)	197 (53.2)	42 (11.4)	62 (16.8)
*p*	<0.001	0.006	<0.001	<0.001	<0.001
**Acute bacterial dysentery**					
Total (n = 73)	60 (82.2)	72 (98.6)	35 (47.9)	24 (32.9)	35 (47.9)
Beijing (n = 43)	34 (79.1)	43 (100)	20 (46.5)	22 (51.2)	33 (76.7)
Shaanxi province (n = 30)	26 (86.7)	29 (96.7)	15 (50.0)	2 (6.7)	2 (6.7)
*p*	0.404	0.855	0.769	<0.001	<0.001

### Management of watery diarrhea and clinical dehydration

Of 554 cases with watery diarrhea overall, 36.6% did not receive fluid and electrolyte replacement; the proportion was significantly higher in Shaanxi province (42.3%) than Beijing (30.4%). Of 132 patients with clinical dehydration, 12.5% did not receive fluid and electrolyte replacement, with no significant difference in this regard between Beijing (13.9%) and Shaanxi province (10.9%).

### Rationality of antibiotic treatment

Antibiotics were considered to be indicated if patients were aged > 65 years or immunocompromised (‘specific cases’), or if patients had invasive diarrhea. In the study, invasive diarrhea include dysentery and patients who temperature > 38.5°C with no stool examination, which according to Wingate’s guidelines for adults on self-medication for the treatment of acute diarrhea [15].

In the overall study group, antibiotics were indicated for 130 (16.3%) patients, but 27 (20.8%) of these did not receive them. Of the 670 patients for whom antibiotics were not indicated, 383 (57.2%) were prescribed antibiotic therapy, accounting for 78.8% of all the patients receiving antibiotics. Overall, 51.3% of patients received irrational antibiotic treatment, comprising 47.9% receiving unnecessary treatment and 3.4% for whom antibiotics were indicated but not prescribed.

## Discussion

While episodes of acute diarrhea are usually brief and self-limiting, treatment can relieve discomfort and other symptoms. Consequently, patients with acute diarrhea often see a physician for medication to relieve their suffering. In the present survey, 31.4% of patients self-medicated before visiting the clinic. The mean interval between the onset of acute diarrhea and going to a specialist hospital diarrhea clinic was 2.4 days. This is similar to data from a study in primary care in Bahrain (2.2 days) [16]. Interestingly, in the present study, the mean interval was shorter in Beijing (2.0 days) than in Shaanxi province (2.8 days). This may reflect the more developed economy in Beijing, such that residents are better able to afford medical expenses, or residents in Beijing may have a greater awareness of the hazards associated with diarrhea.

Fresh stool examination under light microscopy is encouraged for every case of acute diarrhea [17]. However, in the survey, nearly half of all cases have no the examination in Shaanxi province, in Beijing, stool examination was performed in 89.8% of cases. Differences between Beijing and Shaanxi province may reflect regional variations in the importance that physicians attach to stool examination.

In the survey, all patients in Beijing had stool cultures for vibrio cholera. This not only deviates from 2012 WGO guidelines but also does not conform to the national cholera monitoring program. It does reflect local policy in which stool culture for vibrio cholera is required for every patient with acute diarrhea. In light of the discrepancy, it may be appropriate to modify local policy for culture of vibrio cholera.

In general, it is recognized that the principal reason to treat acute episodes of diarrhea is to relieve discomfort and social dysfunction [15]. In the survey, 36.6% of cases with watery diarrhea and 12.5% of cases with clinical dehydration did not receive any fluid and electrolyte replacement; additionally, in Shaanxi province, fewer patients with watery diarrhea received rehydration treatment than those in Beijing (30.4% vs 42.3%). These are clear deviations from 2012 WGO guidelines [6]. The guidelines advise that all adults with watery diarrhea, irrespective of whether they have dehydration, should receive fluid and electrolyte replacement. As the disease is dynamic, and mild dehydration may progress to more severe dehydration, early hydration can prevent fluid deficits [6]. Accordingly, physicians need to improve their knowledge regarding the treatment of diarrhea.

In the study, most patients received ORS as the main rehydration method in Beijing, however, in Shaanxi province, intravenous rehydration was predominated. The pronounced difference was not due to a greater number of patients presenting with vomiting who unable to drink in Shaanxi province, as there was no significant difference between the two regions in this regard. One possible explanation is that physicians in Beijing were all in teaching hospitals those who have more opportunities to learn about the management of acute diarrhea. Another explanation is that intravenous fluid replacement can bring about more income for hospital than would ORS. Besides the financial incentive, the tension relationship between doctors and patients are also the complicated reasons.

In the survey, antibiotics were used in 60.8% of patients, although this therapy was indicated in only 16.3%; and in 38.0% of patients, antibiotics were administered intravenously, even though only 16.3% presented with vomiting who unable to drink. These practices deviate significantly from WGO and national guidelines. Moreover, although physicians in Beijing performed a little better than in Shaanxi province, however, the performance was inconsistent with more opportunities for education available to physicians in Beijing. The extreme misuse of antibiotics in the present survey is similar to that in Thailand [11] and is a very serious and longstanding problem in China [18-20]. More than 20 years ago, 88.9% of patients with acute diarrhea were prescribed antibiotics, with 30.1% having clinically diagnosed bacillary dysentery [20]. More recently, in Guizhou province, diarrhea was still almost universally treated with antibiotics at all levels of medical service [19]. Another recent survey reported that 65% of acute diarrhea patients (both children and adults) were prescribed antibiotic treatment in Guangdong province [20].

Misuse of antibiotics at the community level is also common in China. In the survey, almost one-third of patients self-medicated before visiting a diarrhea clinic, and the most common drugs were, again, antibiotics. This serves as a reminder that nationwide education in the rational use of antibiotics is essential.

In the study, more than half of individuals were prescribed with dioctahedral smectite no matter in Beijing or in Shaanxi although it is currently not recommended by WGO guidelines, reasons for the wider acceptance in these study sites may be due to doctors have more opportunities to learn knowledge of the product and may have also experienced its efficacy and safety for acute diarrhea.

In addition, in the study, in Beijing most patients were prescribed with probiotics and herbal medicines which are also not recommended by WGO guidelines. It is possible their use is driven by favourable prices and ready availability locally. Another reason may be due to they are included by public expenses. In Beijing most patients are included in the public expenses. However, in Shaanxi, less patients are included in the public expense than Beijing.

There were limitations inherent in the study design. This study was a survey of the self-reported practices of physicians in the management of acute diarrhea in adults. Their actual practices may not always be the same as those reported in the questionnaire. The Chinese Standard (2004) for the clinical diagnosis of dysentery, and the guidelines recommended by WGO for the treatment of acute diarrhea in adults, may not always be appropriate due to the complexity of some diarrhea cases, and conflicting data in some studies.

## Conclusion

Tertiary hospital physicians in China do not adhere well to WGO guidelines or to national guidelines for the management of acute diarrhea. These findings suggest that nationwide education and effective health policies are needed to improve medical practice and reduce the unnecessary burden on the healthcare system.

## Abbreviations

ORS: Oral rehydration solution; WGO: World gastroenterology organization.

## Competing interests

The authors have no competing interests to declare.

## Authors’ contributions

FQH, GQW and YL conceived the study and FQH drafted the manuscript. FQH, YW and JL participated in data collection. All authors read and approved the final manuscript.

## Authors’ information

FQH, YW and JL: Associated Chief Physicians, MD, at the Department of Infectious Diseases, Peking University First Hospital, Beijing China.

GQW: Professor, Chief Physician, tutor of postgraduate candidates, and Director of the Department of Infectious Diseases, Peking University First Hospital, Beijing China; Vice-chairman of the Infectious Diseases branch of the Chinese Medical Association and Vice-president of the Infectious Diseases Physicians branch of the Association of Chinese Medical Doctors.

JL: Medical Scientist at the Medial department, Beaufour-Ipsen (Tianjin) Pharmaceutical Co., Ltd, Beijing, China.

## Pre-publication history

The pre-publication history for this paper can be accessed here:

http://www.biomedcentral.com/1471-2458/13/41/prepub
